# Physics-informed deep-learning parameterization of ocean vertical mixing improves climate simulations

**DOI:** 10.1093/nsr/nwac044

**Published:** 2022-03-08

**Authors:** Yuchao Zhu, Rong-Hua Zhang, James N Moum, Fan Wang, Xiaofeng Li, Delei Li

**Affiliations:** CAS Key Laboratory of Ocean Circulation and Waves, Institute of Oceanology, and Center for Ocean Mega-Science, Chinese Academy of Sciences, Qingdao 266071, China; Pilot National Laboratory for Marine Science and Technology (Qingdao), Qingdao 266237, China; University of Chinese Academy of Sciences, Beijing 100049, China; CAS Key Laboratory of Ocean Circulation and Waves, Institute of Oceanology, and Center for Ocean Mega-Science, Chinese Academy of Sciences, Qingdao 266071, China; Pilot National Laboratory for Marine Science and Technology (Qingdao), Qingdao 266237, China; Center for Excellence in Quaternary Science and Global Change, Chinese Academy of Sciences, Xi’an 710061, China; University of Chinese Academy of Sciences, Beijing 100049, China; College of Earth, Ocean and Atmospheric Sciences, Oregon State University, Corvallis, OR 97331, USA; CAS Key Laboratory of Ocean Circulation and Waves, Institute of Oceanology, and Center for Ocean Mega-Science, Chinese Academy of Sciences, Qingdao 266071, China; Pilot National Laboratory for Marine Science and Technology (Qingdao), Qingdao 266237, China; University of Chinese Academy of Sciences, Beijing 100049, China; CAS Key Laboratory of Ocean Circulation and Waves, Institute of Oceanology, and Center for Ocean Mega-Science, Chinese Academy of Sciences, Qingdao 266071, China; CAS Key Laboratory of Ocean Circulation and Waves, Institute of Oceanology, and Center for Ocean Mega-Science, Chinese Academy of Sciences, Qingdao 266071, China; Pilot National Laboratory for Marine Science and Technology (Qingdao), Qingdao 266237, China

**Keywords:** physics-informed deep learning, climate model biases, ocean vertical-mixing parameterizations, long-term turbulence data, artificial neural networks under physics constraint

## Abstract

Uncertainties in ocean-mixing parameterizations are primary sources for ocean and climate modeling biases. Due to lack of process understanding, traditional physics-driven parameterizations perform unsatisfactorily in the tropics. Recent advances in the deep-learning method and the new availability of long-term turbulence measurements provide an opportunity to explore data-driven approaches to parameterizing oceanic vertical-mixing processes. Here, we describe a novel parameterization based on an artificial neural network trained using a decadal-long time record of hydrographic and turbulence observations in the tropical Pacific. This data-driven parameterization achieves higher accuracy than current parameterizations, demonstrating good generalization ability under physical constraints. When integrated into an ocean model, our parameterization facilitates improved simulations in both ocean-only and coupled modeling. As a novel application of machine learning to the geophysical fluid, these results show the feasibility of using limited observations and well-understood physical constraints to construct a physics-informed deep-learning parameterization for improved climate simulations.

## Teaser

Deep learning-based and data-driven parameterizations of ocean vertical mixing under well-defined physical constraints outperform the traditional physics-driven parameterizations, facilitating improved simulations in ocean and climate modeling.

## INTRODUCTION

Climate models serve as powerful tools in climate research. Unfortunately, large and systematic biases remain in all state-of-the-art climate models. One of the largest sources of model biases is related to ocean processes whose spatial scales are smaller than the model grid resolution. Such unsolved subgrid ocean processes must be approximated or ‘parameterized’ in ocean and climate modeling to represent their effects on the processes at resolved scales. Limited by computational resources, current climate models typically resolve ocean processes on horizontal length scales not smaller than 10 km. Ocean turbulence at scales of *O* (10^–2^–10^1^ m) controls thermodynamic mixing of heat, salt, nutrients and other tracers, and greatly affects local and global climates [1–3]. These scales are orders of magnitude smaller than those at which models can resolve, and hence net effects of mixing must be parameterized in numerical models of the coupled ocean-atmosphere system. In particular, turbulent mixing in the tropical oceans is critically important in controlling seasonal sea-surface cooling in the eastern equatorial Pacific and balancing the zonal pressure gradient that drives the equatorial undercurrent (EUC) [4,5]. On the upper flank of the EUC, instability of the sheared flow drives turbulence [6]. Thus, it is not surprising that model biases in the tropical oceans are extremely sensitive to shear-driven mixing parameterizations [7,8]. Instability of a sheared flow depends on the gradient Richardson number (*Ri*; a non-dimensional ratio of stratification, or density gradient, to squared vertical current shear. Strong stratification inhibits instability, whereas strong vertical shear favors instability. Thus, *Ri* defines the instability condition for stratified shear flows). Physics-driven parameterizations based on *Ri* have been employed in many ocean and climate models [9–11]. However, there is large uncertainty in turbulence properties estimated by the *Ri*-based parameterizations. For example, the *K*-profile parameterization (KPP) significantly overestimates the downward turbulent heat flux in the Pacific cold tongue region [12]. Yet this parameterization is widely used in many ocean and climate models; more than one-third of the climate models participating in the Coupled Model Intercomparison Project (CMIP) have adopted KPP, thus increasing uncertainties in CMIP-based climate simulations and projections.

The lack of process understanding primarily causes the poor performance of traditional physics-driven parameterizations. For example, vertical eddy diffusivity (*K_T_*) estimated by *Ri*-based parameterizations monotonically increases with decreasing *Ri*. But different states of flow can exist at the same value of *Ri*, so the parameterized diffusivity is significantly different to observed values [12,13]. In order to reduce uncertainties in vertical-mixing parameterizations, new methods are clearly needed to explore the functional relationship between *K_T_* and resolved oceanic variables. Based on the universal approximation theorem [14,15], it is feasible to parameterize shear-driven mixing using the deep-learning method [16].

Indeed, deep learning has emerged as a powerful data-driven approach to earth science studies [17–19]. For example, this technique has been used for eddy identification [20], El Niño-Southern Oscillation prediction [21] and tropical instability wave forecasting [22]. In recent years, subgrid parameterizations based on deep-learning methods have been investigated [23–25]. In these studies, artificial neural networks learn from high-resolution simulations that have their own subgrid parameterizations. Ultimately, we must directly use observations in deep-learning-based techniques, a significant challenge because *in situ* observations are sparse in both time and space [19,23]; sparse coverage of *in situ* observations can decrease the generalization of deep-learning parameterization (in this study, the ability of a neural network to perform well in the regions where *in situ* observations are absent is called generalization). By introducing physical constraints to neural networks, physics-informed deep learning [26] is a promising approach to addressing this challenge. Thus, this study has developed a novel physics-informed mixing parameterization based on the deep-learning method, which acquires knowledge directly from turbulence observations in the Pacific cold tongue region. To improve the generalization of our parameterization, we used a traditional physics-driven parameterization as a physical constraint on the deep-learning application. Our new parameterization demonstrates good generalization ability, and can improve ocean temperature simulations when employed in ocean-only and coupled climate modeling.

## RESULTS

### Data sources and neural network

Ocean models parameterize turbulent mixing processes in terms of *K_T_* and vertical eddy viscosity (*K_v_*), so that vertical diffusion of tracers and momentum is expressed as ∂*_z_*(*K_ψ_*∂*_z_ψ*), where *ψ* is the tracer concentration or momentum of a fluid parcel. Analogous to a traditional physics-driven parameterization, the task of our parameterization is to predict *K_T_* and *K_v_* as functions of large-scale oceanic variables. The candidate variables that have strong correlations with *K_T_* and *K_v_* may include *Ri*, squared shear *S*^2^, stratification *N*^2^, density *ρ* and velocity *U*. Thus in our first attempt to construct the neural-network-based (NN-based) parameterization, one input vector contains four features [*ρ*, *N*^2^, *U*, *S*^2^]*^T^* (*Ri* is absent from the input features because *Ri* is simply the ratio *N*^2^/*S*^2^). Output variables are *K_T_* and *K_v_*. Compared to direct observations of the input variables, turbulence observations are sparse in time and space. Fortunately, decadal-long time records of turbulence observations [4,27] from the equatorial cold tongues are now available, providing an opportunity to use the neural network to represent the connection between the large-scale ocean state and the shear-driven turbulence. Therefore, temperature *T*(*z*), salinity *SA*(z) and current *U*(*z*) profiles are obtained from the Tropical Atmosphere Ocean (TAO) mooring array [28] at (0^o^, 140^o^W) and the Pilot Research Moored Array in the Tropical Atlantic (PIRATA) [29] at (0^o^, 23^o^W) to calculate the input variables. The output variable *K_T_* is obtained from Oregon State University (OSU) *χ*pod instruments [27] mounted on the TAO and PIRATA moorings (Materials and Methods: Data sources for training the neural network). *K_v_* is not measured by the *χ*pod instruments, thus it is estimated as a function of *Ri* and *K_T_* in our parameterization (Supplementary Data: Parameterization of *K_v_*), consistent with some previous studies [10,11].

In our first attempt to construct the NN-based parameterization, 3400 samples from (0^o^, 140^o^W) are randomly selected to train a fully connected neural network (Fig. S1a). The remaining samples (869 samples from (0^o^, 140^o^W) and all samples from (0^o^, 23^o^W)) are used for validation (Materials and Methods: Training of NN-based parameterization). In general, diffusivities predicted by the NN-based parameterization fit well with the observed ones in the validation data set (Fig. S1b in Supplementary Data online). Before the NN-based parameterization is applied to ocean models, its generalization ability is tested; the full-depth (40–300 m) observations of input variables at (0^o^, 140^o^W) from 2005–2017 are input into the neural network to predict corresponding diffusivities, particularly in the deep oceans where *in situ* turbulence observations are absent. The predicted diffusivities above 150 m agree well with our understanding of shear-driven mixing in the upper equatorial ocean (Fig. S1c). But there exist unrealistically large values of *K_T_* below 150 m, implying that the generalization of the NN-based parameterization must be improved.

### Physical constraint improves the generalization of NN-based parameterization

The failure of our parameterization in predicting *K_T_* below 150 m can be understood from a data science perspective. The data sources for training the neural network are the observations above 119 m and the deeper ocean variables are outside the range of the training data, which presents the key problem in restricting applications of deep-learning parameterizations [23,26]. The pronounced overestimation of *K_T_* can also be understood from a physical oceanographic perspective. Physically, subject to intense deep-cycle turbulence [3,6,30], ocean tracers and momentum are well mixed above the EUC core, leading to a weak stratification and a weak shear in the regions where the training data are measured. In contrast, vertical diffusion is very weak in the ocean interior. Meanwhile, without significant local heat and momentum sources, both stratification and shear are weak in the ocean interior. In such a case, when the NN-based technique is used to predict *K_T_* below the EUC core, our parameterization tends to use the knowledge learned from the regions of deep-cycle turbulence, where a weak stratification and shear corresponds to a large *K_T_*. As a consequence, the predicted *K_T_* is severely overestimated below 150 m.

**Figure 1.**
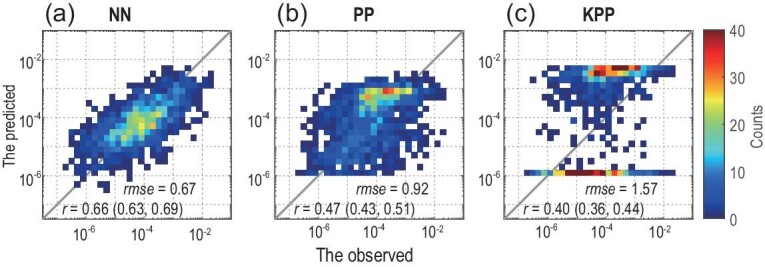
(a–c) The 2D histograms between the observed and the predicted vertical eddy diffusivities. The colors represent the number of data points in each bin. Correlation coefficients (*r*, 95% confidence interval in brackets; *P*-value < 0.001 for all parameterizations) and root-mean-square errors (*rmse*) between the predicted and the observed Log_10_(*K_T_*) are noted in the bottom-right corner. The unit is m^2^ s^–1^.

To improve the generalization of our NN-based parameterization, we incorporated a physical constraint into the neural network to develop a physics-informed deep-learning parameterization (Materials and Methods: Physical constraint; to better understand the methods and terminologies in deep learning, an analogy is provided in Supplementary Data: An analogy for physical constraint). In such a case, an additional parameter, *Ri*, is included in the NN-based parameterization; so the input vector now contains five features [*ρ*, *N*^2^, *U*, *S*^2^, *Ri*]*^T^*, and 850 training samples based on a physics-driven parameterization [10] are added to the training data set (Fig. S2a). After training, the NN-based parameterization is evaluated in the validation data set. Figure 1 shows that the NN-based parameterization represents the observations better than the PP (parameterization proposed by Pacanowski and Philander [10]) and KPP. In addition, the generalization of the NN-based parameterization is greatly improved when a physical constraint is incorporated into the neural network (Fig. S2c). Consistent with our understanding of shear-driven mixing in the equatorial Pacific, the predicted values of *K_T_* are elevated most strongly above the EUC core and less so in the more weakly sheared region beneath the core (also see Fig S4b and c).

**Figure 2.**
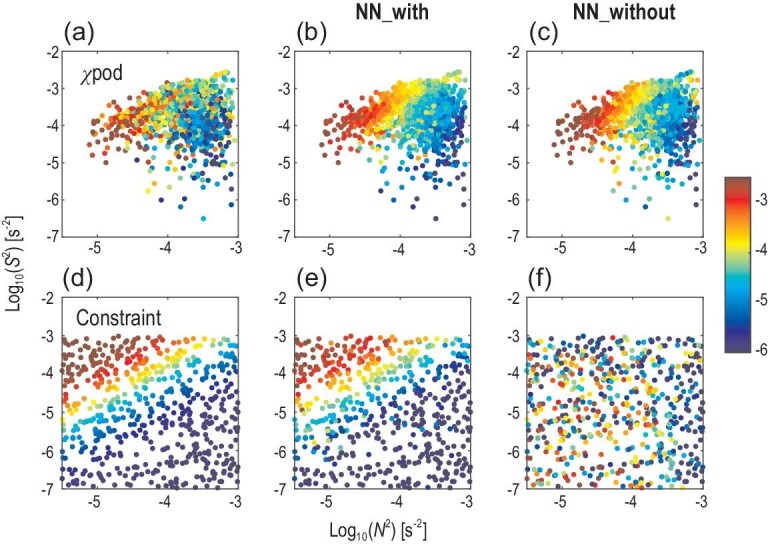
Dependences of *K_T_* on *N* ^2^ and *S* ^2^ in the validation and physics-constraint data sets. The colors represent log_10_(*K_T_*). (a) The observed dependence from *χ*pod instruments. (b and c) The predicted dependence from the NN-based parameterizations with and without physical constraint respectively. (d) The dependence in the physical-constraint data set is based on the PP relation. (e) By adding the physical-constraint data set to training samples, the NN-based parameterization is constrained by a positive correlation between *K_T_* and *S* ^2^ and a negative correlation between *K_T_* and *N* ^2^; (f) these correlations are invalid in the parameterization without physical constraint.

One remaining question is why the NN-based parameterization performs better than the traditional physics-driven parameterizations. Based on the universal approximation theorem [14,15], a feedforward neural network with enough hidden neurons can approximate any function we want to learn. Thus, it is not surprising that the NN-based parameterizations with and without physical constraint can both learn the observed dependences of *K_T_* on *N^2^* and *S^2^* (Fig. 2a–c). However, without physical constraint, the lack of observations under the condition of weak stratification and weak shear (bottom-left corner in Fig. 2a) leads to the predicted *K_T_* in the deep ocean being seriously overestimated (Fig. 2f). That is to say, a better fitting to observations cannot guarantee that neural networks correctly learn the underlying physical mechanisms. Therefore, it is necessary to add a physical constraint to develop an NN-based parameterization that considers the laws of physics (Fig. 2e) and the observational evidence (Fig. 2b).

### Improved simulations in both ocean-only and coupled climate modeling

To assess the performance of the NN-based parameterization, we conduct several numerical experiments, in which the ocean-only modeling is based on the Modular Ocean Model version 5 (MOM5), and the coupled climate modeling is based on the Climate Model version 2.1 (CM2.1) from the Geophysical Fluid Dynamics Laboratory. Shear-driven mixing in the control run is parameterized by KPP (KPP run). For comparison, we also conducted the sensitivity run with the NN-based parameterization (NN run) to test the improved simulations (Supplementary Data: Numerical experiments).

**Figure 3.**
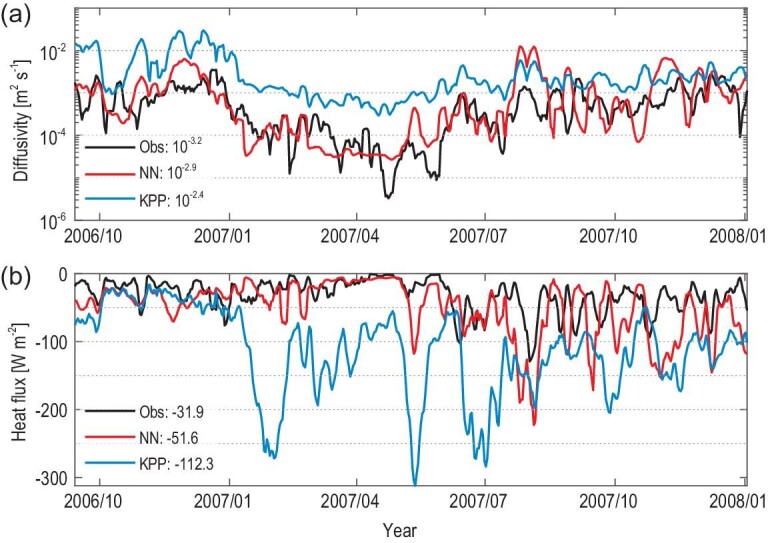
(a and b) Vertical eddy diffusivity and vertical turbulent heat flux in the NN run and in the KPP run. The black lines are the *χ*pod observations at (49 m, (0^o^, 140^o^W); gray shading in Figs S5 and S6); the red lines (NN run) and the blue lines (KPP run) are corresponding simulations from the ocean-only modeling. Time-mean values are noted in the bottom-left corner. The NN run produces a better fit to observations, whereas the KPP run overestimates the *K_T_* and downward (negative) turbulent heat flux.

In the ocean-only modeling, *K_T_* from the NN run exhibits a better agreement with the observation than the KPP run (Figs 3a and S5). Especially above the EUC core, *K_T_* is significantly overestimated in the KPP run but is very close to the observed value in the NN run. The magnitude of *K_T_* directly affects ocean thermal structure through its influence on the vertical turbulent heat flux (*J_q_ = −ρC_p_K_T_T_z_*, where *ρ*, *C_p_* and *T_z_* are the density, heat capacity and vertical derivative of temperature, respectively). Consistent with the overestimated diffusivity, downward turbulent heat flux in the KPP run is generally greater than the observed value over 2005 to 2017 (Figs 3b and S6). In contrast, the vertical turbulent heat flux is more realistic in the NN run.

**Figure 4.**
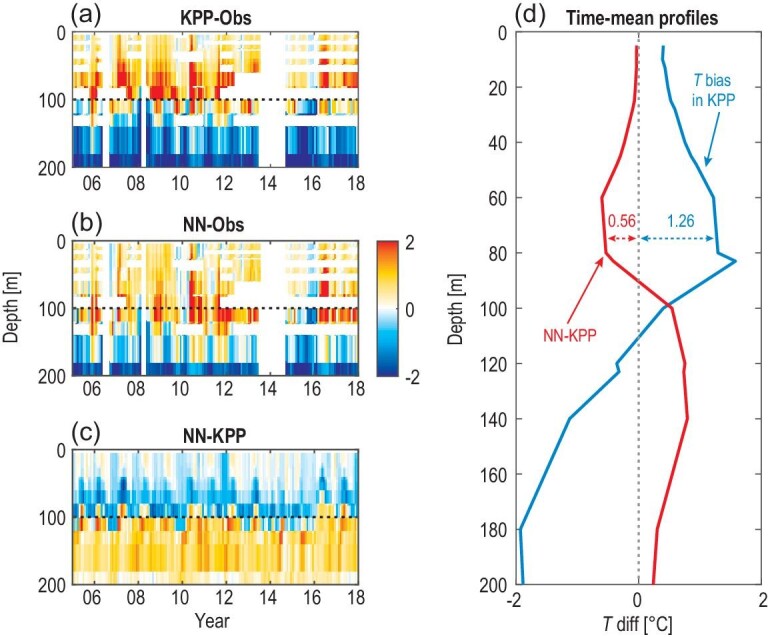
Improved temperature simulations in ocean-only modeling when the NN-based parameterization is implemented into MOM5. (a and b) Temperature bias at (0^o^, 140^o^W) in the KPP run and NN run relative to the TAO observation. (c) The temperature difference between the NN run and the KPP run. Warm bias above and cold bias below ∼120 m can be reduced when the NN-based parameterization is employed. (d) Temperature bias averaged from 2005 to 2017 in the KPP run (blue), and the temperature improvement in the NN run (red). Warm bias between 60 m and 80 m can be reduced by ∼44%. The unit is ^o^C.

The improved simulation of turbulent heat flux provides a more realistic simulation of ocean thermal structure. Figures 4a and S7a demonstrate the temperature bias in the upper equatorial Pacific. In general, the KPP run produces a warm (cold) bias above (below) ∼120 m, which is a typical problem in the ocean-only simulations [31]. Physically, compared with the observation, downward turbulent heat flux is overestimated between 29 m and 69 m but is underestimated at 119 m (Fig. S6b), leading to a heat accumulation above ∼120 m and the consequent warm bias. Meanwhile, the underestimated heat flux is insufficient to heat the ocean layers below 119 m, and the cold bias arises below ∼120 m. The NN run shows a notable improvement in the simulated temperature relative to the KPP run (Figs 4c and d, and S7c). Since the downward turbulent heat flux is more realistic in the NN run (Fig. S6a), the equatorial Pacific temperature bias in the KPP run is greatly reduced.

**Figure 5.**
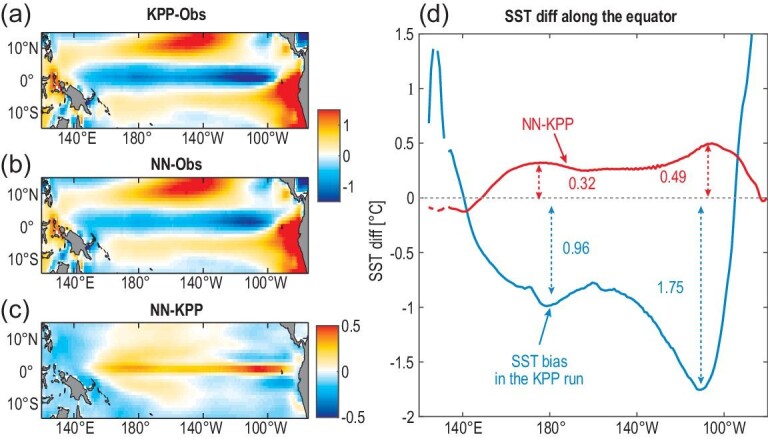
Improved temperature simulations in coupled climate modeling when the NN-based parameterization is implemented into CM2.1. (a and b) Sea surface temperature (SST) bias in the coupled KPP run and NN run relative to Optimum Interpolation SST (OISST) [32] averaged from 1982 to 2017. (c) The SST difference between the NN run and the KPP run. (d) Equatorial SST bias in the KPP run (blue), and the alleviation of cold tongue bias in the NN run (red). The cold SST bias can generally be reduced by ∼30% when the NN-based parameterization is employed. The unit is ^o^C.

It is more challenging to evaluate mixing parameterizations in the coupled simulations because the large-scale oceanic variables treated as input data to the neural network can be poorly simulated due to deficiencies in the atmospheric model. Nevertheless, coupled climate modeling also shows improvements in temperature simulations of the tropical Pacific. The coupled KPP run produces a cold bias in the equatorial upper ocean (Fig. S8a), leading to the well-known Pacific cold tongue bias in climate simulations (Fig. 5a). NN-based parameterization reduces the Pacific cold tongue bias in the coupled KPP run by ∼30% (Fig. 5c and d).

It is worth noting that the PP performs better than the KPP in the validation data set (Fig. 1b and c). Since the PP relation is used to constrain our neural network, the improvements in temperature simulations can be caused by the difference between the PP and the KPP. In order to demonstrate that the improvements are primarily caused by the NN-based parameterization rather than the PP scheme, two additional numerical experiments are conducted. The PP scheme replaces the shear-driven mixing in ocean-only and coupled NN runs. Figures S9 and S10 demonstrate the improved temperature simulations in the NN runs relative to the PP runs. The PP run still has the warm (cold) bias above (below) ∼120 m in the ocean-only simulation (Fig. S9a), and the Pacific too-cold tongue bias in the coupled climate simulation (Fig. S10a). By introducing the NN-based parameterization, temperature bias in the PP runs can also be reduced substantially (Figs S9c and S10c).

## DISCUSSION

The Pacific equatorial cold tongue is a key region whose sea surface temperature (SST) variations impact worldwide through atmospheric teleconnections. It is widely accepted that oceanic turbulent mixing plays a major role in the equatorial heat budget [3,4,30]. However, great uncertainties still exist in the parameterizations of oceanic turbulent mixing [7,8,12,33–35], which has been a primary source for model biases in ocean and climate modeling. Traditional physics-driven parameterizations struggle to explicitly formulate the relationship between large-scale ocean variables and turbulent mixing properties. Unfortunately, the complicated behaviors of ocean turbulent mixing are still not fully understood, and the performance of the corresponding physics-driven parameterizations are not satisfactory. Instead, we have developed a novel parameterization for shear-driven mixing based on a physics-informed deep-learning method in this study. Unlike the traditional physics-driven approach, this data-driven approach learns the underlying relationships directly from the turbulence observations from the Pacific cold tongue. The vertical eddy diffusivity predicted by the NN-based parameterization agrees quite well with the observations, and the NN-based parameterization demonstrates good generalization ability when physical constraint is applied. The feasibility and effectiveness of this NN-based parameterization are further justified by its success in improving ocean temperature simulations in the equatorial Pacific when used in both ocean and coupled climate models.

Parameterizations grounded in theory and tested against observations are an essential part of ocean and climate modeling [36]. However, a lack of theoretical understanding impedes the development of subgrid-scale process parameterizations. Deep learning provides a data-driven approach to parameterizing subgrid processes. An artificial neural network can learn the physical relationships between the unresolved subgrid and resolved large-scale processes without assuming the relational expression in advance. In this way, the NN-based parameterizations can better fit observations than the traditional physics-driven parameterizations. However, the traditional parameterizations based on physical knowledge should not be abandoned completely, and a hybrid physics-informed approach to parameterizations should be considered. Limited by observational technology and funding, observed oceanic variables, such as the vertical eddy diffusivity, have insufficient space-time coverage. These issues decrease the generalization ability of the NN-based parameterizations and cannot be simply solved by adjusting the network structure and parameters. In this case, a constraint on the neural network according to physical knowledge, such as the negative correlation between *K_T_* and *Ri* in this study, would benefit the improved generalization ability of the NN-based parameterizations.

This study applies physical constraint by adding training samples artificially designed based on physical knowledge. In addition, other avenues are very promising—for example, adding a new term representing physical knowledge to loss function or modifying the neural network architectures by adding constraint layers [37,38]. The choice of avenue depends on the accuracy of physical knowledge. In this study, the PP relation is only approximately true, and hence the first two avenues are suitable. But for the neural networks, which must strictly satisfy mass and energy conservation, the latter avenue is more effective.

Many issues remain. Obtaining enough training samples is still key to improving the performance of neural networks. Although many observational programs have shared their turbulence observations generously [2], there are still difficulties in using these observations to improve the accuracy and generalization of the NN-based parameterization. In particular, most of these observations span less than one month, and yield limited data for deep learning. Insufficient data may be partially solved by using large eddy simulations [39] and transfer learning techniques [40]. Specifically, a neural network is trained first on the outputs from large eddy simulations and subsequently on *in situ* turbulence observations. Thus, transfer learning techniques, *in situ* turbulence observations and large eddy simulations should be combined to construct robust parameterizations of oceanic mixing by turbulence. Besides the *Ri*-based parameterizations, two-equation turbulence models are also widely used in ocean general circulation models. In these turbulence models, turbulent kinetic energy (TKE) must be predicted through a prognostic equation, and hence TKE mixing schemes are computationally costly for climate studies. Developing a data-driven TKE mixing scheme with high accuracy and computational efficiency is a promising approach to reducing ocean and climate modeling biases.

## MATERIALS AND METHODS

### Data sources for training the neural network

The data sources for deep learning are from the TAO and PIRATA moorings from 2005 to 2017. For the input features, *U* and *S*^2^ = (d*U*/d*z*)^2^ are calculated using the current profiles observed by Acoustic Doppler Current Profiler (ADCP), *ρ* and *N*^2^ = −(g/*ρ*)(d*ρ*/d*z*) are calculated using the temperature and salinity profiles. Note that there are many missing values in the salinity observations, thus the salinity averaged above 120 m is used to calculate the density. The averaged salinity is 35.2 psu at (0^o^, 140^o^W) and is 36.0 psu at (0^o^, 23^o^W). The use of averaged salinity may degrade the results of our parameterization since the salinity in the eastern equatorial Pacific usually displays a strong vertical gradient. However, our neural network is not very sensitive to the small errors in the training samples. For example, introducing the approximately true PP relation (Materials and Methods: Physical constraint) cannot degrade the accuracy of NN-based parameterization (Figs 1a and S1b). *Ri* = *N*^2^/*S*^2^. As the *χ*pod instruments are mounted separately between 29 m and 119 m at (0^o^, 140^o^W) and between 21 m and 81 m at (0^o^, 23^o^W), the input features are further interpolated to the levels of the *χ*pod measurements. All the input and output variables are daily averaged, and 4269 samples at (0^o^, 140^o^W) and 663 samples at (0^o^, 23^o^W) are obtained.

### Training of NN-based parameterization

The neural network is a fully connected network with three hidden layers (Figs S1a and S2a); this architecture can provide the best performance in the validation data set (Fig. S3). The input vectors contain four or five features, each of which is normalized to zero mean and unit variance. The output variable is the base-10 logarithm of *K_T_*_._ During the training, the LeakyReLU activation function is used to produce the output of hidden neurons, and the Adam optimizer is used to minimize the squared error between the predicted and the observed log_10_(*K_T_*). Using the TensorFlow library [41], the neural network is trained for 2 × 10^4^ epochs. Development of the NN-based parameterization is completed after the training.

### Physical constraint

In order to improve the generalization of our NN-based parameterization, physical constraint is incorporated into the neural network. First, *Ri* is added as the fifth feature of the input vector (Fig. S2a). Second, 850 samples (named the physical-constraint data set) physically produced based on the PP relation [10]

$$\begin{equation*}
{K_T} = \frac{{\frac{{5 \times {{10}^{ - 3}}}}{{{{\left( {1 + 5Ri} \right)}^2}}} + {{10}^{ - 4}}}}{{1 + 5Ri}} + {10^{ - 6}}\,\,{\rm{ }}{\rm {m}^2}\,\,{\rm{ }}{{\rm{s}}^{ - 1}}
\end{equation*}$$

are added to the training data. Specifically, in one physically produced sample, *ρ*, *N*^2^, *U* and *S*^2^ are randomly selected between 1022–1045 kg m^–3^, 10^–6^–10^–3^ s^–2^, −0.4–1.2 m s^–1^ and 10^–8^–10^–3^ s^–2^, respectively. *Ri* is calculated from *N*^2^/*S*^2^. The corresponding *K_T_* is calculated based on the PP relation. In this way, we design 850 samples serving as the training data. In other words, 20% of the training data (850/(3400 + 850)) provide the physical constraint that *K_T_* is negatively correlated with the *Ri*. It is obvious that the NN-based parameterization with the physical constraint can predict the observed diffusivities rather well (Fig. S2b and c).

## Supplementary Material

nwac044_Supplemental_File
