# Supplementary material for: Physics-informed deep-learning parameterization of ocean vertical mixing improves climate simulations
Source: Natl Sci Rev. 2022 Mar 8;9(8):nwac044. doi: 10.1093/nsr/nwac044 (PMC9385460; doi:10.1093/nsr/nwac044)
Supplement: nwac044_Supplemental_File [file nwac044_supplemental_file.docx]

**Supplementary Data for**

**Physics-informed Deep Learning Parameterization of Ocean Vertical Mixing Improves Climate Simulations**

Yuchao Zhu^1, 2, 5^, Rong-Hua Zhang^1, 2, 4, 5*^, James N. Moum^3^, Fan Wang^1, 2, 5^, Xiaofeng Li^1^, and Delei Li^1, 2^

^1^CAS Key Laboratory of Ocean Circulation and Waves, Institute of Oceanology, and Center for Ocean Mega-Science, Chinese Academy of Sciences, Qingdao 266071, China.

^2^Pilot National Laboratory for Marine Science and Technology (Qingdao), Qingdao 266237, China.

^3^College of Earth, Ocean and Atmospheric Sciences, Oregon State University, Corvallis, Oregon 97331, USA.

^4^Center for Excellence in Quaternary Science and Global Change, Chinese Academy of Sciences, Xi’an 710061, China.

^5^University of Chinese Academy of Sciences, Beijing 100049, China.

*Corresponding author. **Email:** rzhang@qdio.ac.cn

**This file includes:**

Supplementary text

Figures S1 to S10

**Supplementary text**

**An analogy for physical constraint.** To better understand the methods and terminologies in deep learning, an analogy example is provided below.

We have thousands of photos of dogs and cats. Using these photos, we trained a robot that can accurately distinguish between cats and dogs.


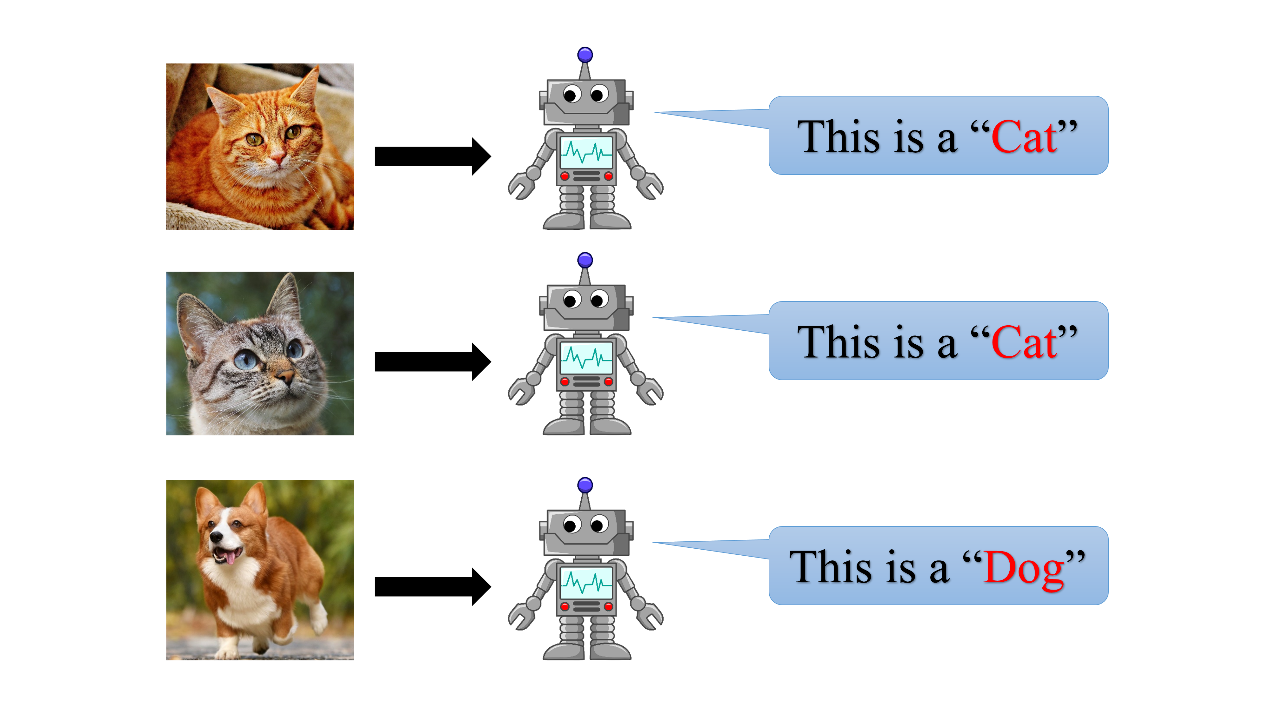


But someone wants to fool this robot. He gives the robot a photo of an elephant. This is beyond the capabilities of the robot because it has never seen an elephant. In this case, it will randomly identify the elephant in this photo as a cat or a dog.


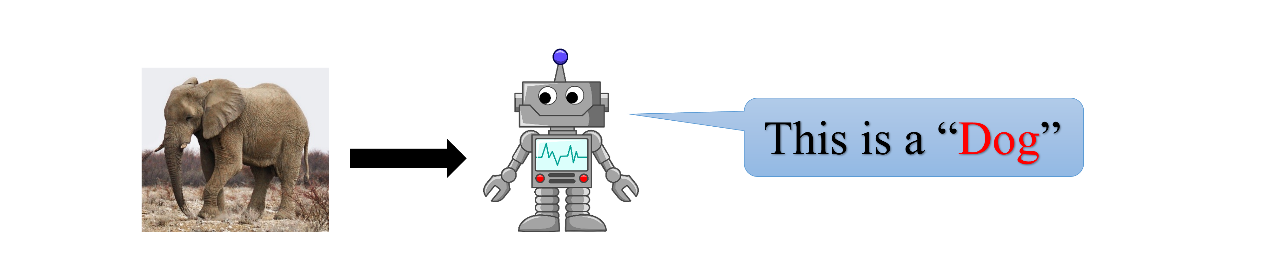


It is the same difficulty we had in this study. We have thousands of samples measured in the upper ocean (above 129 m), and we use these samples to train a neural network (Fig. S1a) that can realistically predict the vertical eddy diffusivity in the upper ocean (Fig. S1b). We want to test (or “fool”) the neural network by evaluating its performance in the deep ocean (below 129 m). Unsurprisingly, it fails to predict the vertical eddy diffusivity in the deep ocean (Fig. S1c) because it has never seen the samples in the deep ocean. In other words, the ***generalization ability*** of the robot (neural network) is poor.

Let's go back to the example of the robot. We want to improve the generalization ability of this robot. Unfortunately, we do not even have a photo of an elephant (We only have thousands of photos of dogs and cats). So this robot cannot learn to recognize elephants the way it learns to distinguish between cats and dogs. To overcome this issue, we need a constraint. Specifically, I am good at painting. I draw a lot of pictures of elephants, and tell the robot “They are elephants”.


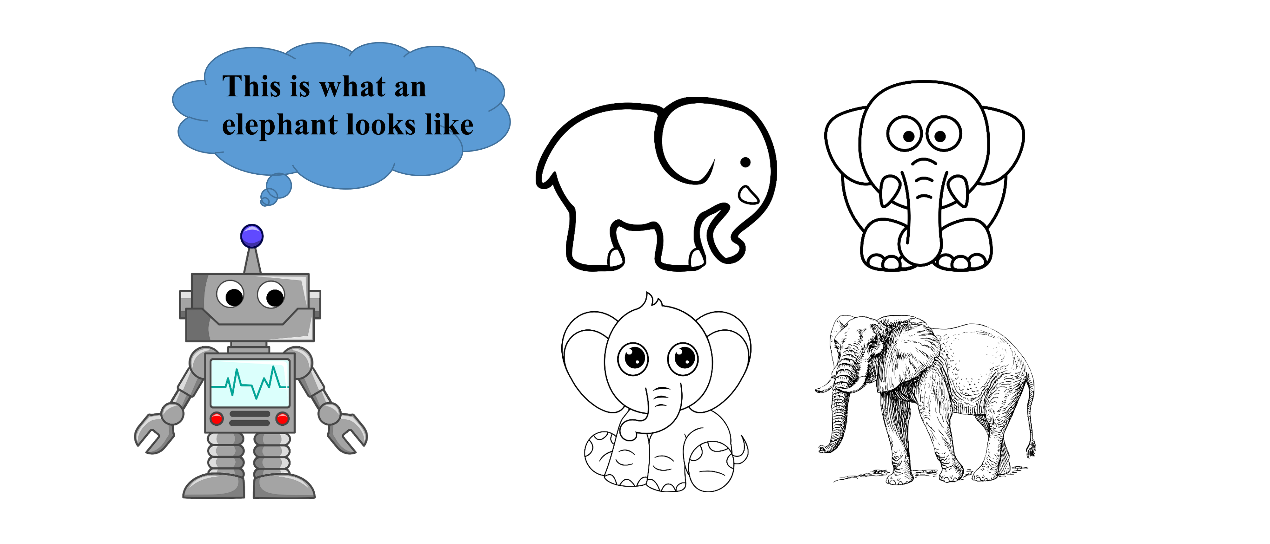


It is a fact that there are some differences between the elephant I drew and the true elephant. Nevertheless, some important features can be learned by the robot, such as the long nose, the big ears, and so on. In this way, the robot can recognize elephants.


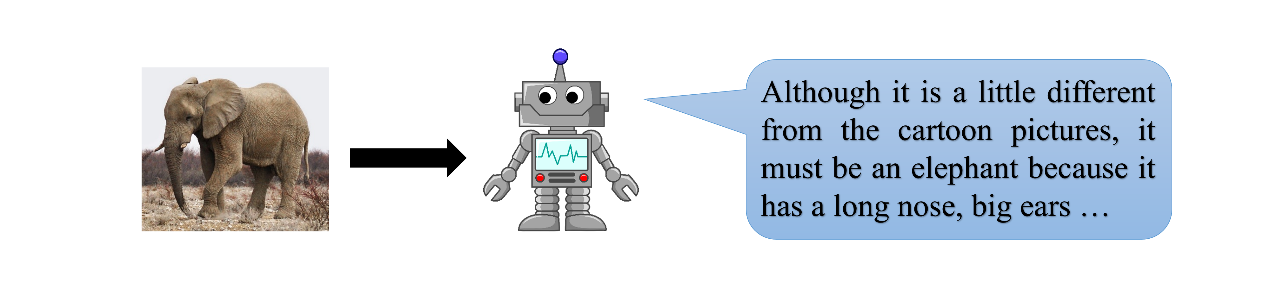


In our study, in order to improve the generalization of our NN-based parameterization, a constraint is incorporated into the neural network. Since this constraint is based on a physics-driven parameterization (PP relation), we call it a ***physical constraint***. Specifically, using the PP relation, we designed 850 artificial samples. It is a fact that there is large uncertainty in the PP relation. Nevertheless, some important features can be learned by the neural network, such as the negative correlation between *K_T_* and *Ri*. In this way, the NN-based parameterization can predict the vertical eddy diffusivity in the deep ocean (Fig. S2c).

In summary:

| **the example of a robot** | **counterparts in our study** |
| --- | --- |
| the robot | the NN-based parameterization |
| thousands of photos of dogs and cats | the observed *K_T_* above 129 m |
| the photos of elephants (absent) | the observed *K_T_* below 129 m (absent) |
| the cartoon elephants I drew | artificially designed 850 samples using the PP relation (physical constraint) |
| the elephant should have a long nose and big ears (Not always correct, for example, Asian elephants have small ears) | negative correlation between *K_T_* and *Ri* (Not always correct, for example, different states of flow can exist at the same value of *Ri*) |
| the robot can recognize elephants | the NN-based parameterization can predict the *K_T_* in the deep ocean more realistically |
| The generalization ability is improved. | |

**Parameterization of *K_v_*.** The ratio of vertical eddy viscosity to vertical tracer diffusivity is defined by the turbulent Prandtl number *P_r_*=*K_v_*/*K_T_*. For the stably stratified shear flows, *P_r_* is close to 1 at low *Ri*, and increases with increasing *Ri*. Thus, different empirical parameterizations based on the *Ri* have been proposed. For example, *P_r_*=0.8+3*Ri* based on the large eddy simulation [1,2]; *P_r_*=1+5*Ri* based on the microstructure observations in the eastern tropical Pacific [3]. In this study, *P_r_*=min (1+5*Ri*, 10) is used not only because it is from the observations in the tropical ocean, but also because it has been employed by many ocean general circulation models [4].

**Numerical experiments.** In order to test the improved performances of the NN-based parameterization, MOM5-based [5] ocean-only simulations are conducted in the ocean modeling. The MOM5 has a horizontal resolution that is 1^o^ in longitude and varies from 1/3^o^ at the equator to 1^o^ in high latitude, and 50 vertical levels with 10 m resolution in the upper 220 m. In the control run (KPP run), KPP is used to parameterize the boundary layer mixing, shear-driven mixing and double-diffusive mixing. In the sensitivity run (NN run), the shear-driven mixing component of KPP

$$K_{T}=\left\{\begin{matrix} 5\times10^{-3}\text{+}10^{-6}\text{ }Ri\leq0 \\ 5\times10^{-3}\left[1-\left(Ri/0.8\right)^{2}\right]^{3}\text{+}10^{-6}\text{ }0<Ri\leq0.8 \\ 10^{-6}\text{ 0.8}<Ri \end{matrix}\right.\text{ }m^{2}\text{ }s^{-1}$$

is replaced by the NN-based parameterization in the tropical Pacific (10^o^S-10^o^N, 120^o^E-80^o^W). Two runs are initialized using the observed January temperature and salinity, and are integrated for 59 years using the JRA55-do forcing fields [6] from 1959 to 2017. Model outputs for the last 13 years (2005–2017) are saved for analysis. In the climate modeling, CM2.1-based [7] coupled simulations are conducted. The ocean component is MOM5 with the identical configurations to the ocean-only simulations. The atmospheric component AM2.1 has a horizontal resolution of 2.0^o^ latitude by 2.5^o^ longitude with 24 vertical levels, and the horizontal resolution of land component LM2.1 is the same as that of the AM2.1. The ocean, atmospheric and land components exchange fluxes every 2 hours without flux adjustments. Similar to the ocean-only simulations, the control run in the climate modeling uses the shear-driven mixing component of KPP, whereas the sensitivity run uses the NN-based parameterization for the shear-driven mixing. The coupled experiments are integrated for 150 years using the values of greenhouse gases, aerosols, isolation, and land cover in 1990, and the analysis is based on the outputs for the last 50 years.

**Data and materials availability.** TAO and PIRATA mooring data are available at https://www.pmel.noaa.gov/tao/drupal/disdel/, https://www.pmel.noaa.gov/tao/drupal/chipod/index.html, https://cchdo.ucsd.edu/cruise/TAO, and https://cchdo.ucsd.edu/cruise/PIRATA. The NOAA OISST is available at https://www.ncei.noaa.gov/products/optimum-interpolation-sst. The EN4 dataset is available at https://www.metoffice.gov.uk/hadobs/en4. The code for the NN-based parameterization can be downloaded at https://doi.org/10.5281/zenodo.5637576.

**
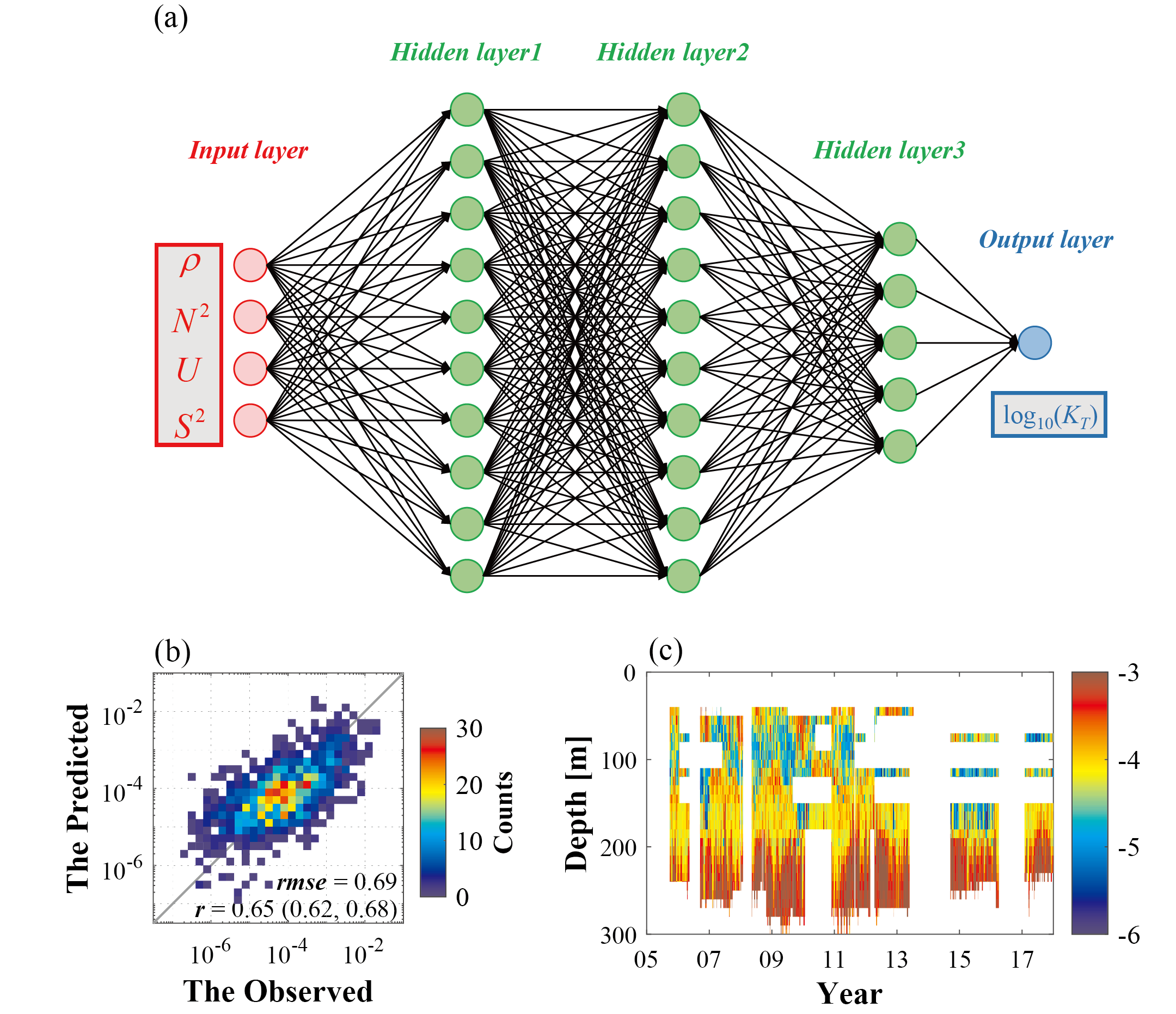
**

**Figure S1.** Structure, validation and generalization of the NN-based parameterization. (a) The neural network is a fully connected network with 3 hidden layers (green). At first, there are only 4 features (red) in one input vector [*ρ*, *N*^2^, *U*, *S*^2^]*^T^*. The output variable is the base-10 logarithm of the *K_T_* (blue). (b) Comparison between the observed and the predicted *K_T_* in the validation dataset. (c) The log_10_(*K_T_*) at (0^o^, 140^o^W) predicted by the NN-based parameterization. In order to test the generalization of the NN-based parameterization, the temperature, salinity and current profiles in the entire upper ocean (40-300 m) at (0^o^, 140^o^W) during 2005-2017 are used to calculate the input vectors, and subsequently to predict the corresponding log_10_(*K_T_*). It is obvious that the large diffusivities below 150 m are inconsistent with our understanding of the shear-driven mixing in the deep ocean. This is why the physical constraint must be incorporated into the neural network to improve the generalization of our parameterization. The unit is m^2^ s^-1^.

**
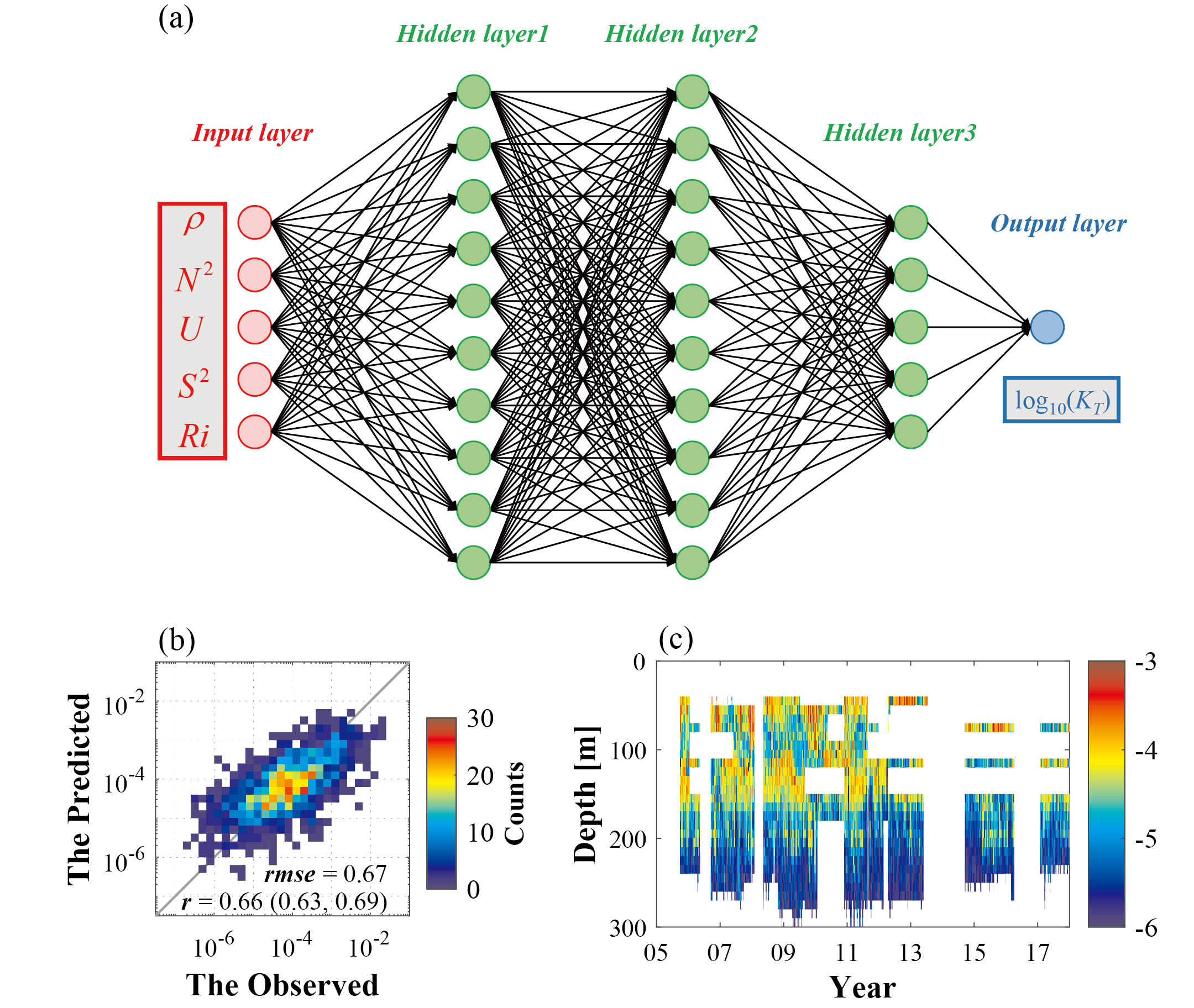
**

**Figure S2.** As in Fig. S1, but for the NN-based parameterization with physical constraint. Compared with that in Fig. S1a, the neural network contains 5 input features. In the validation dataset, the performances of the NN-based parameterizations with and without physical constraint are similar. However, the generalization is greatly improved when the physical constraint is incorporated into the neural network, and the shear-driven mixing is only elevated on the flanks of the EUC (also see Figs. S4b and S4c), which is consistent with our understanding of the shear-driven mixing in the equatorial Pacific. The unit is m^2^ s^-1^.


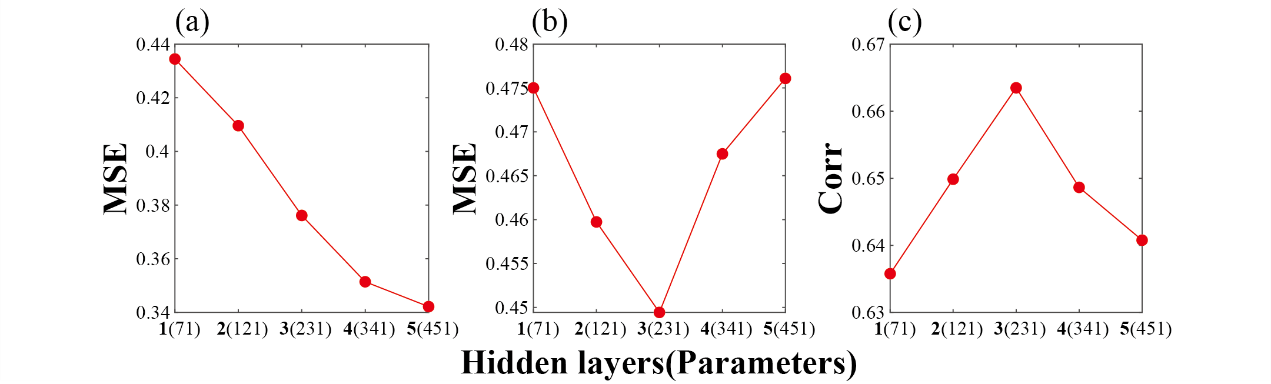


**Figure S3.** The mean square error (MSE) and correlation (Corr) between the predicted and the observed Log_10_(*K_T_*) using the different neural network architectures. X-axis is the number of hidden layers and trainable parameters (in brackets). (a) MSE in the training dataset. (b and c) MSE and Corr in the validation dataset. It is shown that the neural network architecture with 3 hidden layers produces the smallest MSE and the highest Corr.


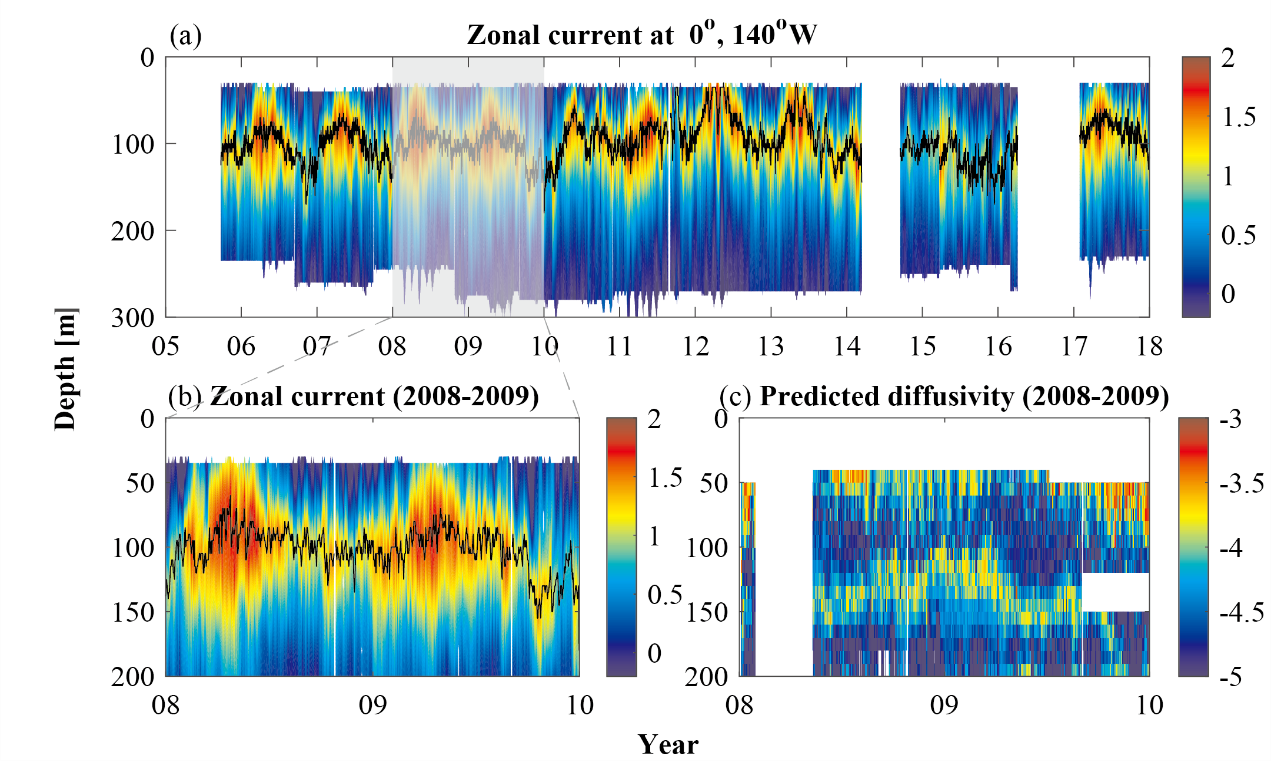


**Figure S4.** Vertical structure of the EUC at (0^o^, 140^o^W). (a) Zonal current from 2005 to 2017. The core of the EUC (eastward velocity maximum; the black line) is located near 100 m. Thus, the upper flank of the EUC will refer to the region between 30 m and 100 m. (b) A two-year sample of zonal current, and (c) the corresponding log_10_(*K_T_*) predicted by the NN-based parameterization (also see Fig. S2c). The unit is m s^-1^ in (a) and (b), and is m^2^ s^-1^ in (c).


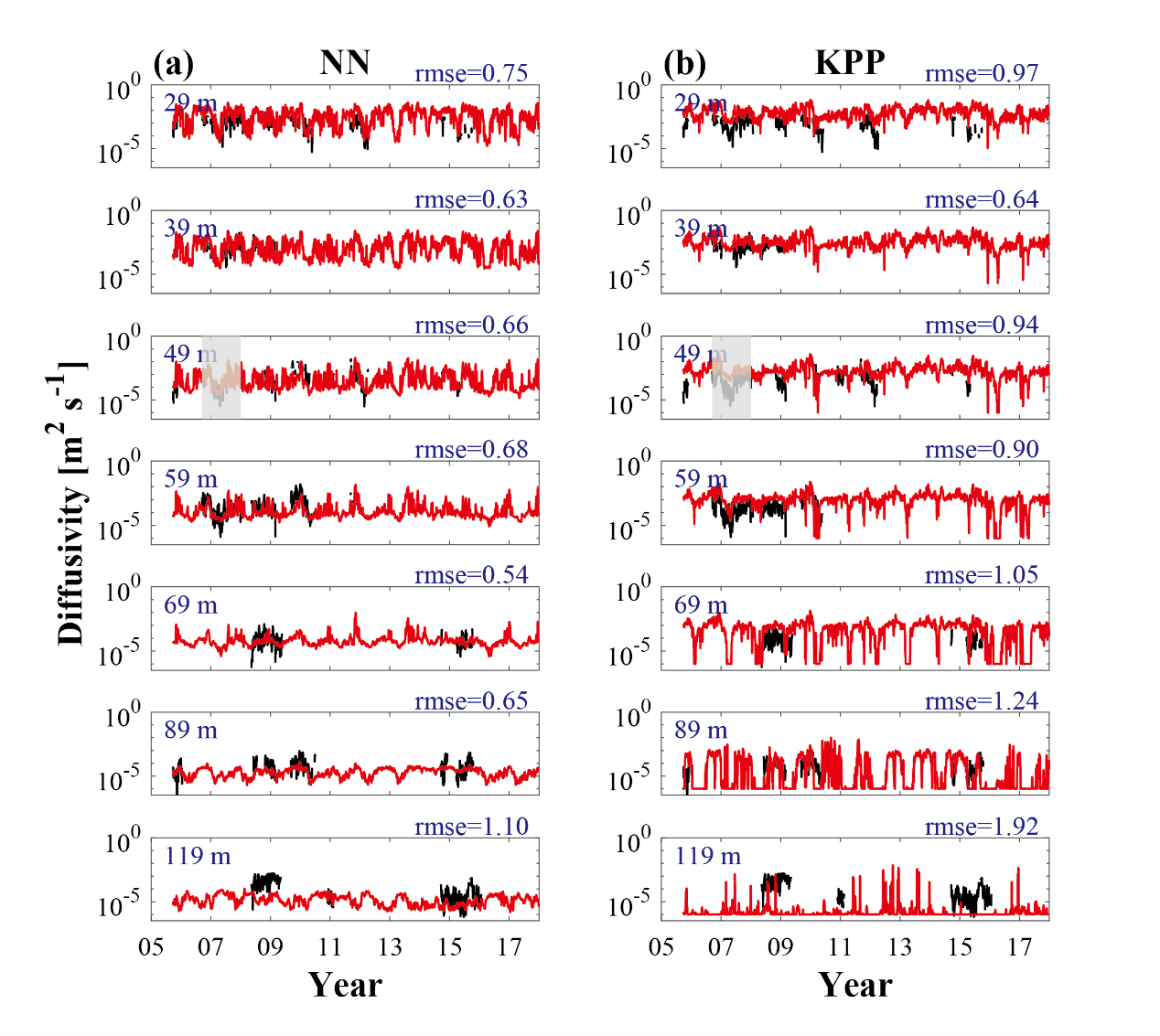


**Figure S5.** Vertical eddy diffusivities in the NN run and in the KPP run. The black lines are the *χ*pod observations at (0^o^, 140^o^W), and the red lines are the simulations from the ocean-only modeling. It is shown that the NN run produces a better fit to observations than the KPP run. Especially on the upper flank of the EUC (29-69 m), vertical eddy diffusivity is overestimated in the KPP run.


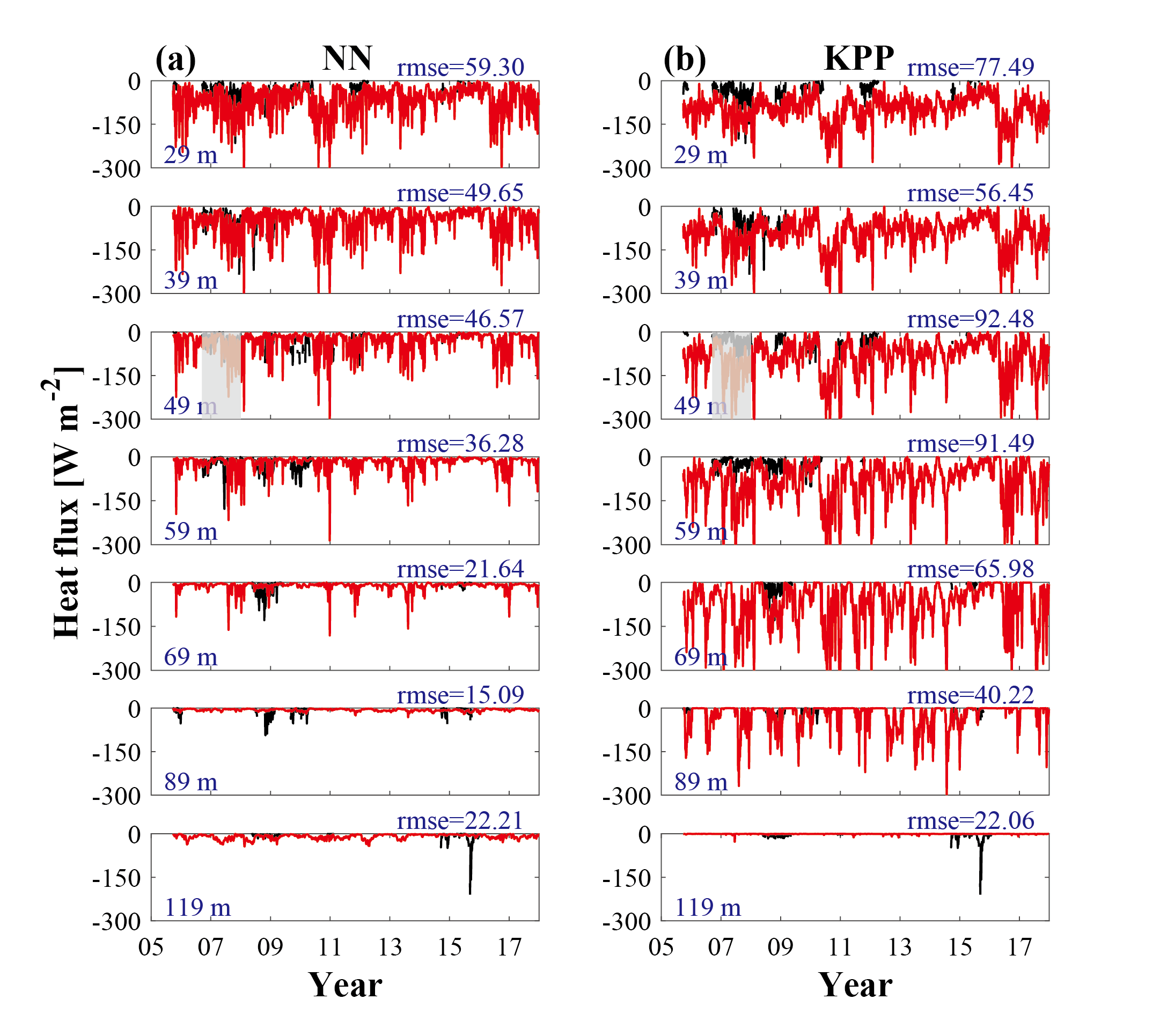


**Figure S6.** As in Fig. S5, but for the vertical turbulent heat flux in the NN run and in the KPP run. Consistent with the results in Fig. S5, the KPP run generally produces an overly strong downward (negative) turbulent heat flux. But the turbulent heat flux is more realistic in the NN run.


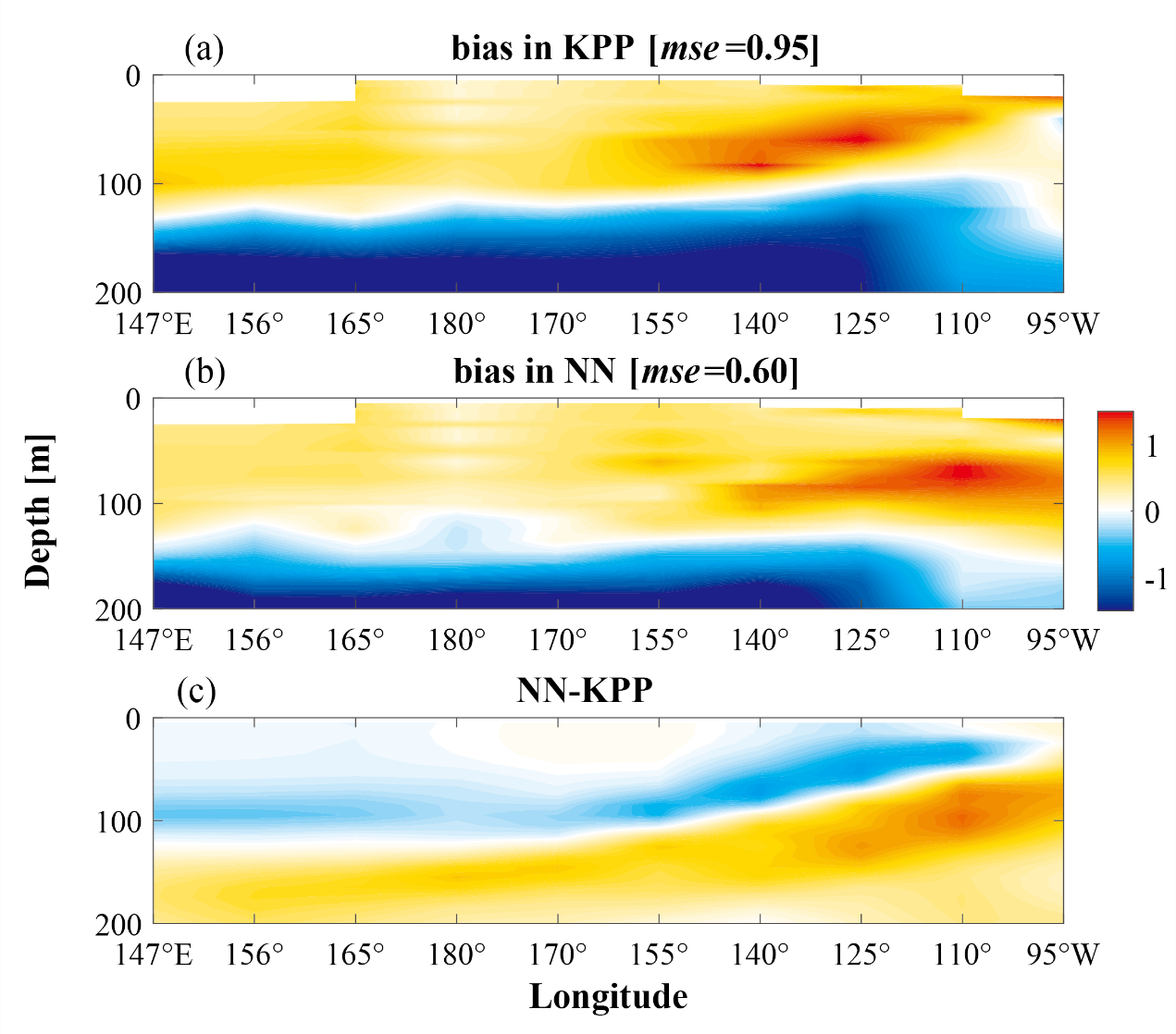


**Figure S7.** Equatorial Pacific temperature bias in the ocean-only modeling. (a and b) The equatorial temperature bias averaged from 2005 to 2017 relative to the TAO observation in the KPP run and the NN run. The mean square error is shown in the title. (c) The temperature difference between the NN run and the KPP run. The unit is ^o^C.


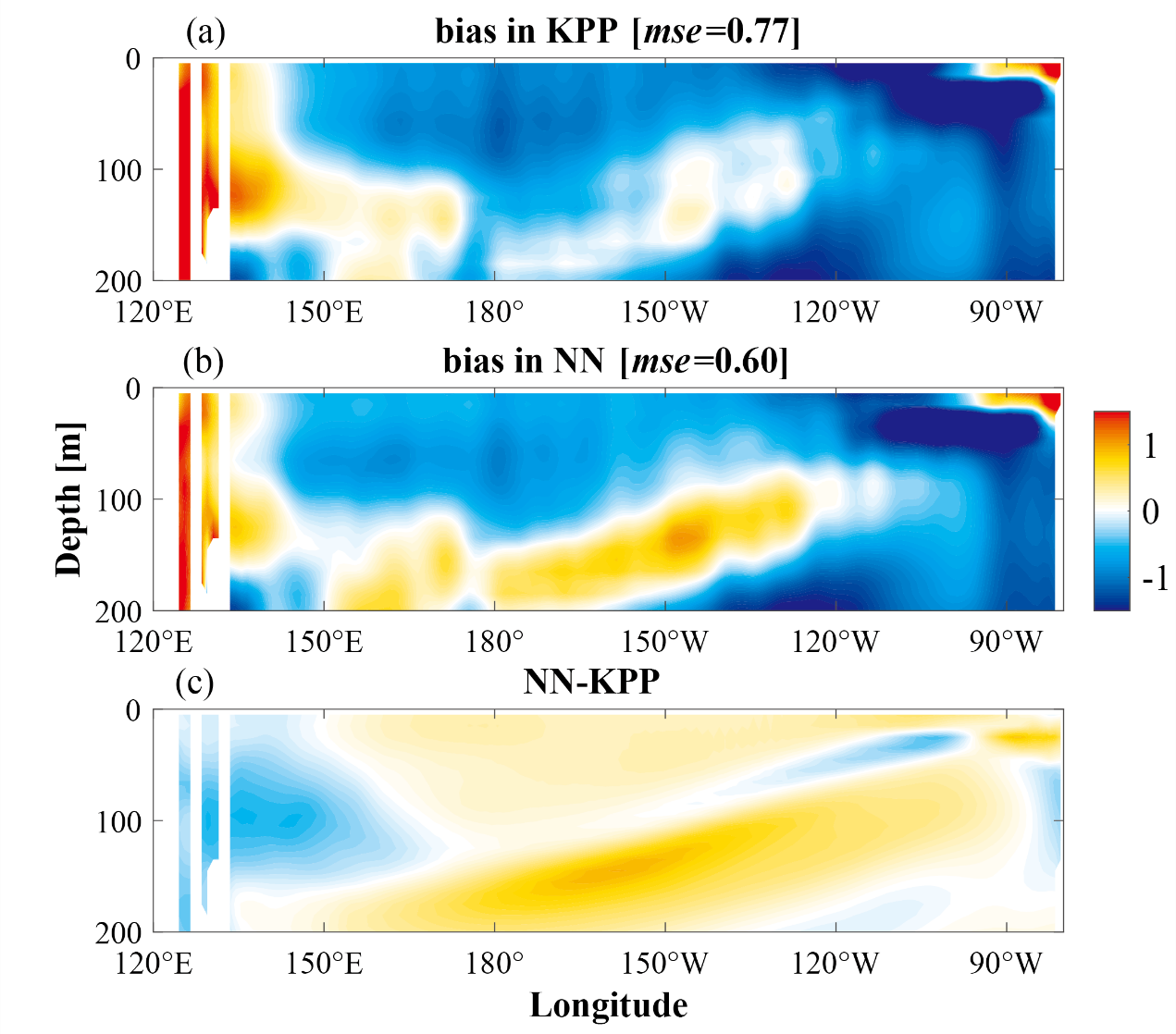


**Figure S8.** Equatorial Pacific temperature bias in the coupled climate modeling. (a and b) The equatorial temperature bias relative to EN4 [8] in the KPP run and the NN run. The mean square error is shown in the title. Considering the CM2.1 is driven by the constant forcing fields in 1990, the version 4 of the Met Office Hadley Centre “EN” series of data sets (EN4) from 1982-2017 are temporally averaged to calculate the observed temperature along the equator. The simulated equatorial temperature is calculated by averaging the model output over the entire 50 years. (c) The temperature difference between the NN run and the KPP run. The unit is ^o^C.


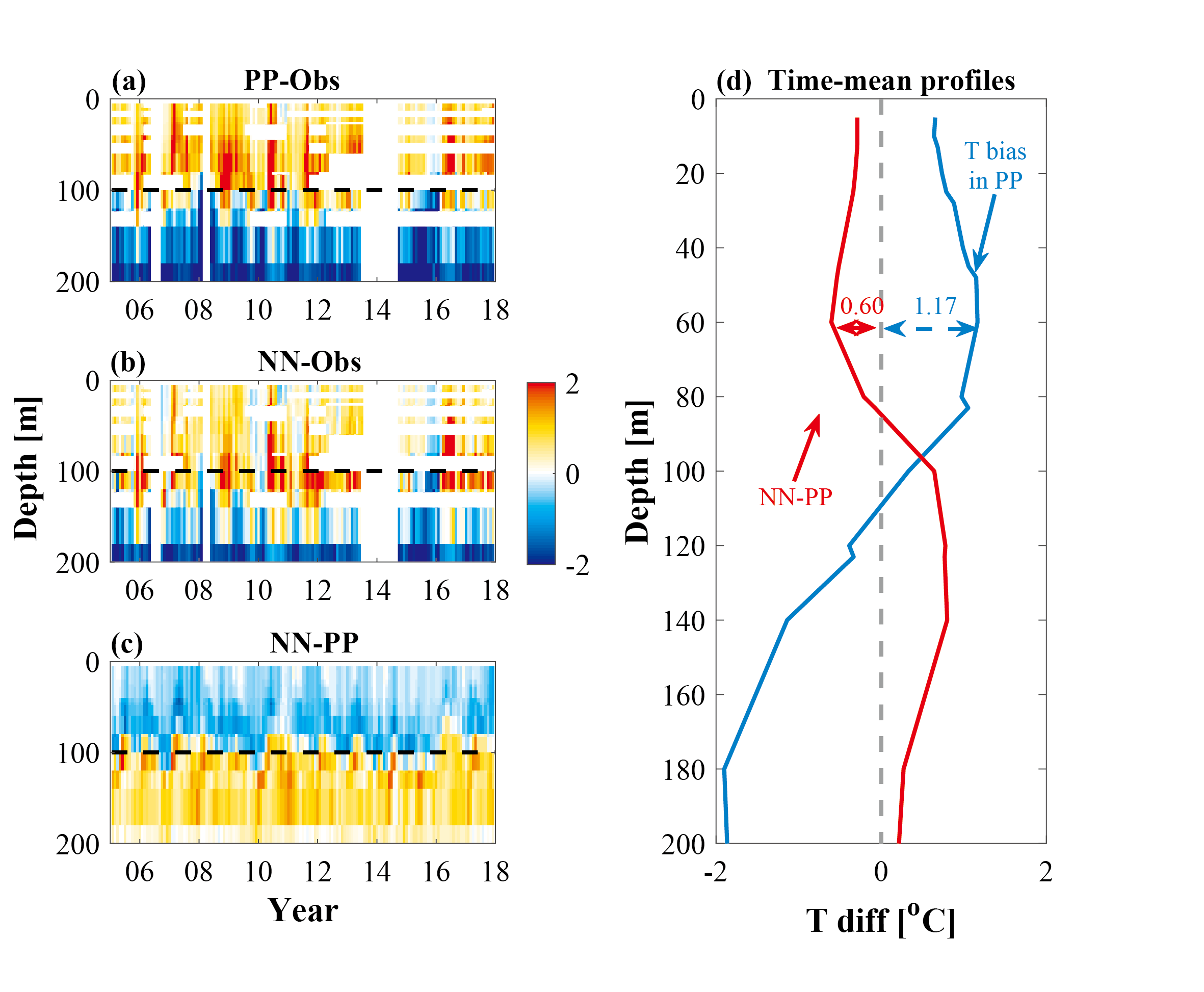


**Figure S9.** As in Fig. 4, but for the temperature differences between the ocean-only PP run and NN run.


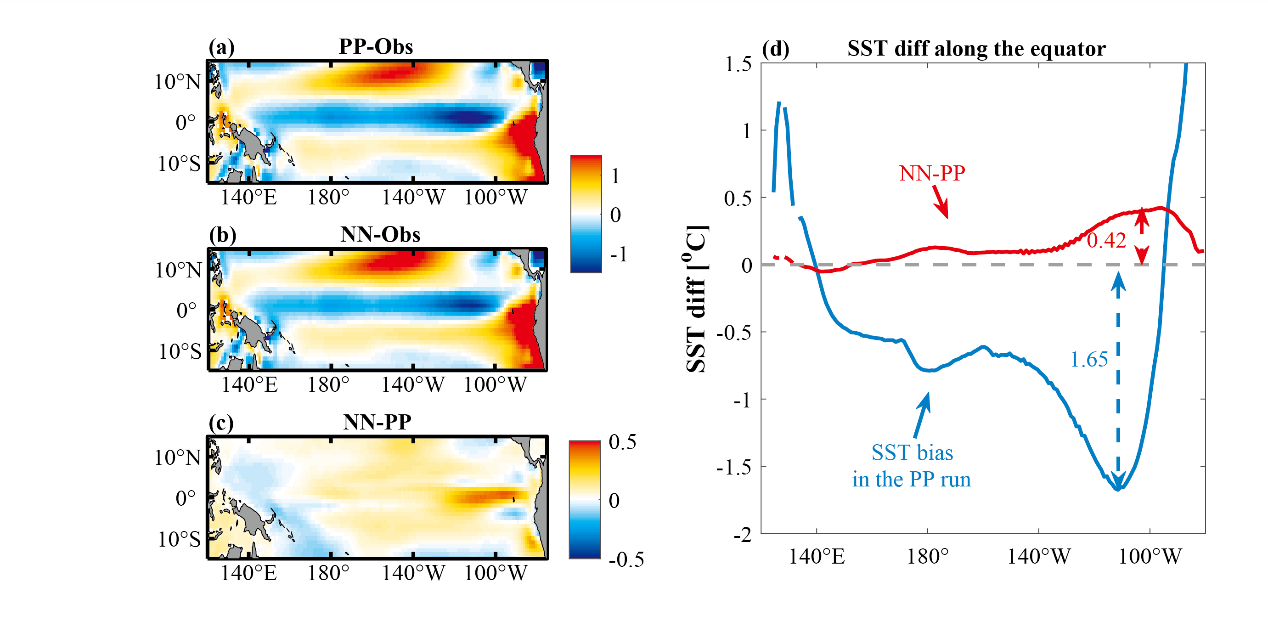


**Figure S10.** As in Fig. 5, but for the temperature differences between the coupled PP run and NN run.

**References**

1. Esau IN and Grachev AA. Turbulent Prandtl number in stably stratified atmospheric boundary layer: Intercomparison between LES and SHEBA data. *e-WindEng* 2007; **5**: 1-17.

2. Smyth WD, Nash JD and Moum JN. Self-organized criticality in geophysical turbulence. *Sci Rep* 2019; **9**: 3747.

3. Peters H, Gregg MC and Toole JM. On the parameterization of equatorial turbulence. *J Geophys Res* 1988; **93**: 1199-218.

4. Pacanowski RC and Philander SGH. Parameterization of Vertical Mixing in Numerical Models of Tropical Oceans. *J Phys Oceanogr* 1981; **11**: 1443-51.

5. Griffies SM. Elements of the modular ocean model (MOM). *GFDL Ocean Group Tech Rep* 2012; **7**: 620.

6. Tsujino H, Urakawa S and Nakano H *et al.* JRA-55 based surface dataset for driving ocean–sea-ice models (JRA55-do). *Ocean Modell* 2018; **130**: 79-139.

7. Delworth TL, Broccoli AJ and Rosati A *et al.* GFDL's CM2 Global Coupled Climate Models. Part I: Formulation and Simulation Characteristics. *J Climate* 2006; **19**: 643-74.

8. Good SA, Martin MJ and Rayner NA. EN4: Quality controlled ocean temperature and salinity profiles and monthly objective analyses with uncertainty estimates. *J Geophys Res* 2013; **118**: 6704-16.
